# Machine-learning based prediction of prognostic risk factors in patients with invasive candidiasis infection and bacterial bloodstream infection: a singled centered retrospective study

**DOI:** 10.1186/s12879-022-07125-8

**Published:** 2022-02-13

**Authors:** Yaling Li, Yutong Wu, Yali Gao, Xueli Niu, Jingyi Li, Mingsui Tang, Chang Fu, Ruiqun Qi, Bing Song, Hongduo Chen, Xinghua Gao, Ying Yang, Xiuhao Guan

**Affiliations:** 1 Key Laboratory of Immunodermatology, Department of Dermatology, The First Hospital of China Medical University, No.155 Nanjing Bei Street, Heping District, Shenyang, 110001 Liaoning Province People’s Republic of China; 2 Department of Dermatology, Integrated Hospital of Traditional Chinese Medicine, Southern Medical University, Guangzhou, 510315 People’s Republic of China; 3 Department of Dermatology, The First Affiliated Hospital of Sun Yat-Sen University, 510080 Guangzhou, People’s Republic of China; 4 Department of Biotechnology, Beijing Institute of Radiation Medicine, 27 Taiping Road, Haidian District, Beijing, 100850 People’s Republic of China; 5 School of Dentistry, Cardiff University, Heath Park, Cardiff, CF14 4XY UK

**Keywords:** Bacterial bloodstream infection, China, Epidemic, Invasive candidal infection, Machine learning

## Abstract

**Background:**

Invasive candidal infection combined with bacterial bloodstream infection is one of the common nosocomial infections that is also the main cause of morbidity and mortality. The incidence of invasive Candidal infection with bacterial bloodstream infection is increasing year by year worldwide, but data on China is still limited.

**Methods:**

We included 246 hospitalised patients who had invasive candidal infection combined with a bacterial bloodstream infection from January 2013 to January 2018; we collected and analysed the relevant epidemiological information and used machine learning methods to find prognostic factors related to death (training set and test set were randomly allocated at a ratio of 7:3).

**Results:**

Of the 246 patients with invasive candidal infection complicated with a bacterial bloodstream infection, the median age was 63 years (53.25–74), of which 159 (64.6%) were male, 109 (44.3%) were elderly patients (> 65 years), 238 (96.7%) were hospitalised for more than 10 days, 168 (68.3%) were admitted to ICU during hospitalisation, and most patients had records of multiple admissions within 2 years (167/246, 67.9%). The most common blood index was hypoproteinemia (169/246, 68.7%), and the most common inducement was urinary catheter use (210/246, 85.4%). Moreover, the most frequently infected fungi and bacteria were *Candida parapsilosis* and *Acinetobacter baumannii*, respectively. The main predictors of death prognosis by machine learning method are serum creatinine level, age, length of stay, stay in ICU during hospitalisation, serum albumin level, C-Reactive protein (CRP), leukocyte count, neutrophil count, Procalcitonin (PCT), and total bilirubin level.

**Conclusion:**

Our results showed that the most common candida and bacteria infections were caused by Candida parapsilosis and *Acinetobacter baumannii*, respectively. The main predictors of death prognosis are serum creatinine level, age, length of stay, stay in ICU during hospitalisation, serum albumin level, CRP, leukocyte count, neutrophil count, PCT and total bilirubin level.

**Supplementary Information:**

The online version contains supplementary material available at 10.1186/s12879-022-07125-8.

## Background

Invasive candidal infection and bacterial bloodstream infections are common in hospitals [1, 2]. It is reported that approximately 1.5 million patients with invasive candidal infection die every year worldwide [3, 4]. Bacterial bloodstream infection is the seventh leading cause of death in North America and Europe [5]. In addition, in these regions, the average annual mortality is 29 cases per 100,000 population, and the total mortality is between 13 and 20% [5]. However, the epidemiological characteristics of both invasive candidal infection and bacterial bloodstream infections differ with geographic location and time [6–12]. In the past two decades, the incidence of non-*Candida albicans* infection has increased. A recent study from Japan revealed that *Candida albicans* was the infectious agent in 58.2% of all candidiasis cases in 2003 but only in 30% of cases in 2014. In addition, a study from China reported that *Candida albicans* was the causative agent in only 44.9% of invasive candidal infection cases [13], which is consistent with our previous study, which revealed that *Candida albicans* is no longer the most common invasive fungus [14]. The risk of death owing to invasive fungal and bacterial bloodstream infections puts enormous pressure on healthcare services, leading to a shortage of intensive care resources. However, in previous studies, the epidemiological characteristics and risk factors of invasive fungal infections complicated with bacterial bloodstream infections were rarely discussed, possibly owing to the limited capacity of some methods in analysing large datasets.

Machine learning techniques have the unique ability to deal with extensive data because they can process large datasets in a flexible and trainable manner and understand the complex relationship between variables [15]. Owing to their improved processing ability, various machine learning and artificial intelligence techniques are widely used to identify risk and prognostic factors of disease in patients to help clinicians. Therefore, we conducted a retrospective analysis of patients with invasive candidal infection concomitant with bacterial bloodstream infection and identified the prognostic indicators of death using machine learning methods.

## Methods

### Patient selection

Patients were selected as previously described [14]. We collected all data on *Candida*, *Cryptococcus* and other yeast isolates recovered from the blood, ascitic fluid, peritoneal dialysate fluid, pus and tissues of patients with invasive candidal infection (2008 version of EORTC/MSG criteria). The onset of bacterial bloodstream infection or invasive candidal infection was defined as the date when the first positive result of blood culture was obtained. The data collected included patient characteristics at baseline, haematological diagnoses and chemotherapy, risk factors for invasive candidal infection, clinical features of invasive candidal infection, *Candida* test results, bacterial test results, antifungal prophylaxis and treatment and survival status at discharge. In addition, data regarding the management of patients receiving antifungal prophylaxis or therapy were recorded, including the date and nature of the change in treatment and survival status at discharge. The hospitalisation of each patient represented one event, and if a patient was re-hospitalised and received another round of treatment, he/she was considered a new event. Persistent candidal infections was defined as persistent if positive blood culture results were obtained for the same *Candida* species 7 days after the initiation of appropriate antifungal therapy [16]. We excluded non-*Candida* yeast samples and samples from non-sterile sources, such as faeces, urine, sputum, pharyngeal swabs and pus.

### Microbiological tests

Aseptic humoral samples (8–10 mL) were collected and cultured for 5 days. Samples with positive results were transferred to blood agar plates, and subsequently, bacterial and fungal isolates were cultured at 35 °C for 48–72 h. Gram staining and microscopic examination were performed simultaneously. Strains (bacterial and fungal isolates) were identified on a VITEK 2 Compact system (Bio-Merieux SA, Marcy l ‘etoile, France), and susceptibility tests were performed using the ATB FUNGUS 3 kit (Bio-Merieux SA, Marcy l ‘etoile, France).

The minimum inhibitory concentration (MIC) was determined according to the CLSI m27-a3 and m27-s4 antifungal susceptibility test standards. The quality control strains used were *Candida* ATCC6258 and *Candida albicans* ATCC90028.

### Machine learning methods

We pre-processed the data and deleted missing cases with 50% features, and the mean value of missing values was filled. The dataset was randomly divided into the training and test sets (7:3), with 70% patients in the training set and 30% patients in the test set. We used the random forest, logistic regression and support-vector machine algorithms to build a prediction model. Subsequently, the trained random forest model was analysed to evaluate the feature importance ranking.

### Statistical analysis

The IBM SPSS Statistics for Windows version 20.0 software (IBM Corp., Armonk, NY, USA) was used for statistical analysis. Non-normally distributed quantitative data were expressed as median and quartile ranges [M (P25, P75)] and analysed using the Mann–Whitney test for intergroup comparisons. Qualitative data were represented by relative numbers, and the chi-square test was used for intergroup comparisons.

### Definition and abbreviations

ICU, intensive care unit; SDD, susceptible-dose-dependent; PCT, procalcitonin; CRP, C-reactive protein; BDG, 1-3-β-d-glucan.

Prolonged hospitalisation was defined as hospital stay longer than 10 days. Surgery was defined as thoracic and abdominal surgeries. Recent surgery was defined as surgery performed 14 days before the first diagnosis of *Candida* infection. Abdominal surgery was defined as any surgery involving organs including the stomach, small intestine, colon or rectum, gallbladder, liver, pancreas, spleen and appendix. Concerning laboratory results, renal failure was defined as creatinine clearance < 60 mL/min, hypoalbuminaemia was defined as serum albumin concentration < 30 g/L and leukopaenia was defined as peripheral white blood cell count < 4 × 10^9^cells/L. Prolonged ICU stay was defined as ICU stay for more than 10 days. Long-term and combined use of multiple antibiotics were defined as the use of antibiotics for more than 14 days and the simultaneous use of more than 2 antibiotics, respectively. Multiple bacterial infections were defined as infections with more than two types of bacteria simultaneously. Multiple fungal infections were defined as infections with more than two types of fungi simultaneously.

## Results

### Clinical features of patients

A total of 246 patients with invasive candidal infection complicated with bacterial bloodstream infection were included in this study. The median age of the patients was 63 years (53.25–74 years). Among the 246 patients, 159 (64.6%) were men, 109 (44.3%) were aged more than 65 years, 238 (96.7%) were hospitalised for more than 10 days, 168 (68.3%) had been admitted to ICU during hospitalisation and 167 (67.9%) had multiple admission records within the past 2 years. The common concomitant conditions of patients with invasive candidal infection complicated with bacterial bloodstream infection were hypoproteinaemia (169/246, 68.7% patients), surgery within the past 2 weeks (112/246, 45.5% patients), solid tumours (96/246, 39.0% patients), septic shock (58/246, 23.6% patients), diabetes (47/246, 19.1% patients), renal failure (36/246, 14.6% patients) and pancreatitis (25/246, 10.2% patients). Other common risk factors included the use of urinary catheter (210/246, 85.4% patients), central venous catheter (185/246, 75.2% patients), gastric tube (166/246, 67.5% patients), drainage catheter (168/246, 68.3% patients), invasive mechanical ventilation (153/246, 62.2% patients) and total parenteral nutrition (196/246, 79.7%). In addition, long-term use (181/246, 73.6%) and combined use of multiple antibiotics (162/246, 65.9%) were common in patients with invasive candidal infection complicated with bacterial bloodstream infection. Detailed data can be found in Additional file 1: Table S1.

**Fig. 1:**
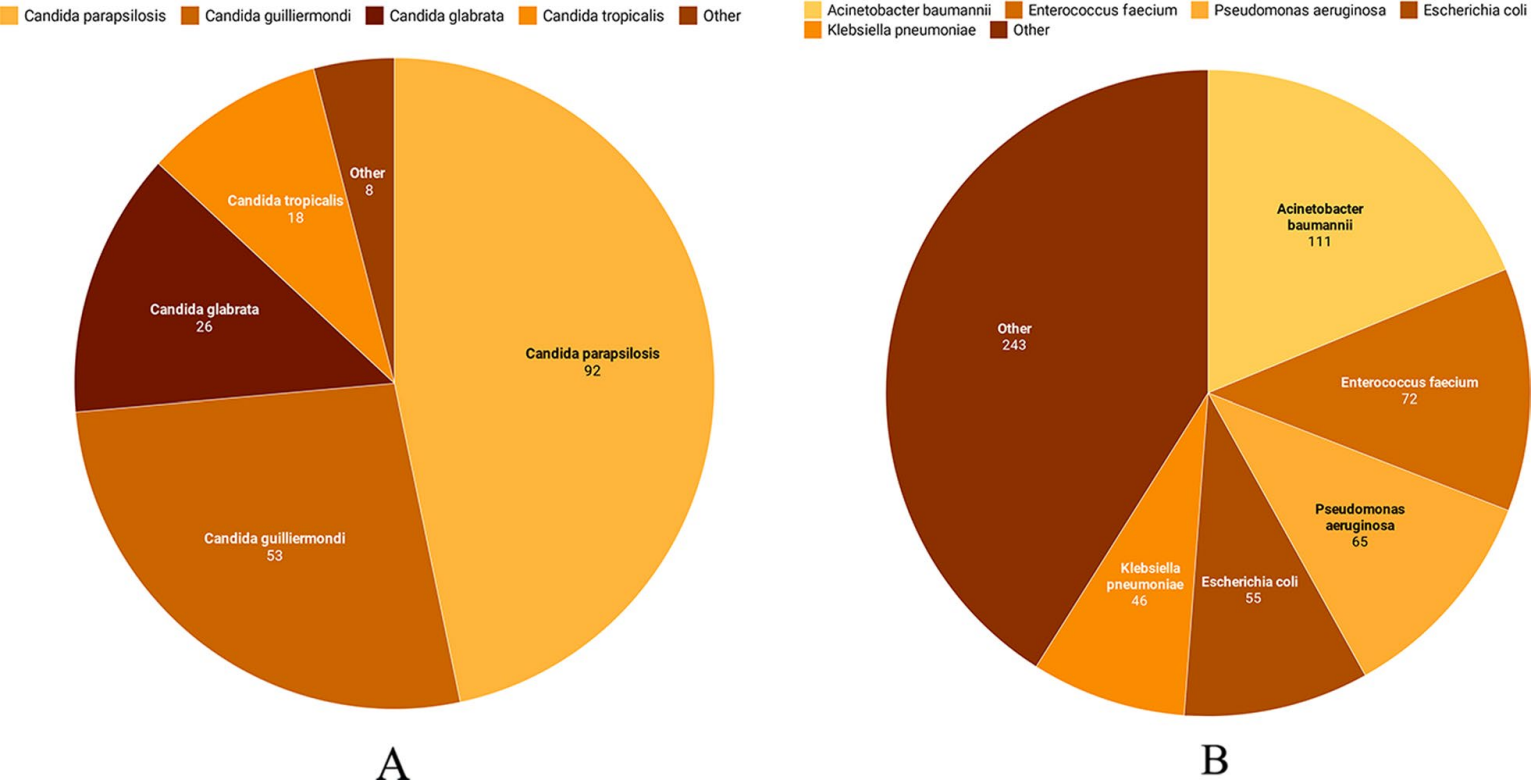
Distribution of the pathogens found in the 246 hospitalized patients. **A** The most frequently infected fungi are *Candida parapsilosis* (92/246, 37.4%), followed by *Candida guilliermondi* (53/246, 21.5%), *Candida albicans* (49/246, 19.9%), *Candida glabrata* (26/246, 10.6%), *Candida tropicalis* (18/246, 7.3%), *Candida krusei* (4/246, 1.6%), *Candida lusitaniae* (2), *Candida streptococcus* (1), and *Cryptococcus neoformans* (1). **B** The most common bacterias infecting the patients were *Acinetobacter baumannii* (111/246, 45.1%), *Enterococcus faecium* (72/246, 29.3%), *Pseudomonas aeruginosa* (65/246, 26.4%), *Escherichia coli* (55/246, 22.4%), and *Klebsiella pneumoniae* (46/246, 18.7%)

The most common causative agent was *Candida parapsilosis* (infecting 92/246, 37.4% patients), followed by *Candida guilliermondi* (53/246, 21.5% patients), *Candida albicans* (49/246, 19.9% patients), *Candida glabrata* (26/246, 10.6% patients), *Candida tropicalis* (18/246, 7.3% patients), *Candida krusei* (4/246, 1.6% patients), *Candida lusitaniae* (2), *Candida streptococcus* (1) and *Cryptococcus neoformans* (1) (Fig. 1A).

Furthermore, 28 species of bacteria were isolated; of which, 15 (53.6%) were Gram-positive and 13 (46.4%) were Gram-negative. Moreover, *Acinetobacter baumannii* (111/246, 45.1% patients) was the most common causative agent of bacterial bloodstream infection, followed by *Enterococcus faecium* (72/246, 29.3% patients), *Pseudomonas aeruginosa* (65/246, 26.4% patients), *Escherichia coli* (55/246, 22.4% patients) and *Klebsiella pneumoniae* (46/246, 18.7% patients) (Fig. 1B). In addition, there were 73 (29.7%) cases of single and 173 (70.3%) cases of multiple bacterial bloodstream infections. Detailed data can be found in Additional file 2: Table S2.

### In vitro antifungal susceptibility test

We obtained 239 isolates from 246 patients with drug sensitivity; of which, 19 (7.9%) isolates were resistant to at least one antifungal agent. Amphotericin B showed excellent results, as all strains were sensitive to it. The drug sensitivity of voriconazole and fluorouracil was good, achieving an efficiency of 96.6% (230/238) and 98.3% (235/239), respectively. In addition, the drug sensitivity of fluconazole and itraconazole was 85.8% (205/239) and 90.4% (216/239), respectively. *Candida glabrata* isolates were highly susceptible to fluconazole (18/26, 69.2%) and itraconazole (10/26, 38.5%) in a dose-dependent manner. *Candida tropicalis* isolates exhibited considerable resistance to fluconazole (5/17, 29.4%) and voriconazole (5/17, 29.4%), whereas *Candida krusei* isolates exhibited strong resistance to fluconazole (3/4, 75%). Detailed data can be found in Additional file 3: Table S3.

### Risk factors for *Candida albicans* and non-*Candida albicans* infections

The demographic and clinical characteristics of patients with *Candida albicans* and non-*Candida albicans* infections are shown in Table 1. It was found that 34.69% patients with *Candida albicans* infection had diabetes; however, only 15.23% patients with *candidiasis* caused by a different *Candida* species had diabetes. Moreover, 81.63% patients with *Candida albicans* infection were admitted to ICU as opposed to 64.97% patients with *candidiasis* caused by a different *Candida* species. In addition, 65.31% patients with *Candida albicans* infection and 83.25% patients with *candidiasis* caused by a different *Candida* species were administered parenteral nutrition. Regarding catheterisation, 74.62% patients with catheter drainage had non-*albicans* candidiasis, whereas 42.86% patients with catheter drainage were infected with *Candida albicans*. In addition, patients with diabetes or those admitted to ICU had a higher risk of contracting *Candida albicans* infection, whereas parenteral nutrition and catheterisation increased the risk of non-*albicans candidiasis*. Differences were statistically significant.

**Table 1:** Risk factors for *Candida albicans* and non-*Candida albicans* infections

	*Candida albicans* % (n = 49)	Non-*Candida albicans* % (n = 197)	Statistic	*P value*
Male	31 (63.27%)	128 (64.97%)	0.050	0.823
Age (years)^a^	63.00 (56.00, 79.00)	63.00 (53.00, 74.00)	1.013	0.311
Length of stay (days)^a^	40.00 (24.00, 75.00)	49.00 (32.00, 73.00)	− 1.205	0.228
Length of stay in ICU^a^	9.00 (5.00, 22.00)	10.00 (0.00, 30.00)	0.404	0.686
Solid tumor	17 (34.69%)	79 (40.10%)	0.482	0.487
Diabetes	17 (34.69%)	30 (15.23%)	9.620	0.002
Pancreatitis^b^	4 (0.82%)	21 (10.66%)	–	0.793^b^
Total parenteral nutrition	32 (65.31%)	164 (83.25%)	7.807	0.005
Renal failure	9 (18.37%)	27 (13.71%)	0.683	0.409
Recent surgery (within 2 weeks)	22 (44.90%)	90 (45.69%)	0.010	0.921
Use immunosuppressants within the past 30 days^b^	6 (12.24%)	9 (4.57%)	–	0.086^b^
Stay in ICU during hospitalization	40 (81.63%)	128 (64.97%)	5.029	0.025
Hypoproteinemia	37 (75.51%)	132 (67.01%)	1.320	0.251
Invasive mechanical ventilation	32 (65.31%)	121 (61.42%)	0.337	0.561
Urinary catheter	41 (83.67%)	169 (85.79%)	0.140	0.708
Gastric tube	28 (57.14%)	138 (70.05%)	2.979	0.084
Central venous catheter	32 (65.31%)	153 (77.66%)	3.214	0.073
Drainage catheter	21 (42.86%)	147 (74.62%)	18.282	< 0.001
Septic shock	14 (28.57%)	44 (22.34%)	0.847	0.357
Multiple hospitalizations within 2 years (> 2 times)	34 (69.39%)	133 (67.51%)	0.064	0.801
Persistent fungal infection	28 (57.14%)	110 (55.84%)	0.027	0.869
Serum albumin level^a^ (g/L)	26.10 (23.40, 29.90)	27.45 (23.40, 31.00)	− 0.435	0.664
Serum creatinine level^a^ (μmol/L)	75.00 (47.00, 103.00)	57.50 (39.75, 86.25)	2.230	0.026
Leukocyte count^a^ (10^9^/L)	10.15 (7.72, 14.47)	7.68 (5.29, 10.86)	3.730	< 0.001
Total bilirubin level^a^ (μmol/L)	14.90 (8.90, 25.70)	15.30 (9.70, 28.60)	− 0.635	0.526
Neutrophil count^a^ (10^9^/L)	8.26 (5.42, 12.39)	6.32 (4.06, 8.95)	3.038	0.002
Lymphocyte count^a^ (10^9^/L)	0.77 (0.52, 1.21)	0.83 (0.56, 1.18)	− 0.068	0.945
CRP^a^ (mg/L)	127.50 (94.63, 186.28)	98.10 (62.78, 143.75)	2.802	0.005
PCT^a^ (ng/mL)	1.34 (0.51, 8.25)	0.56 (0.23, 2.09)	3.049	0.002

^a^Is described by median and quartile, and the statistic was the Z value; other items were described as numbers (n—%) and the statistic was the χ^2^ value

^b^Statistic was the Fisher χ^2^ value

Furthermore, the C-reactive protein (CRP) and procalcitonin (PCT) levels were markedly elevated in patients with invasive candidal infection complicated with bacterial bloodstream infection, especially in those with *Candida albicans* infection. In addition, the CRP and PCT levels were higher in patients with *Candida albicans* infection than in patients with non-*Candida albicans* infection, and the difference was statistically significant. However, both leukocyte and lymphocyte counts were within the normal range. Detailed data are provided in Table 1.

### Analysis of risk factors in patients with persistent and non-persistent *Candida* infections

The demographic and clinical characteristics of patients with persistent and non-persistent *Candida* infection are shown in Table 2. Persistent *Candida* infection was associated with diabetes, longer stay in the ICU and renal failure. Differences were statistically significant.

**Table 2:** Risk factors in patients with persistent and non-persistent candidal infections

	Persistent candidal infection (%) (n = 138)	Non-persistent candidal infection (%) (n = 63)	Statistic	*P value*
Male	88 (63.31%)	39 (61.90%)	0.065	0.799
Age (years)^a^	64.50 (54.00, 74.00)	61.00 (52.50, 70.50)	1.324	0.185
Length of stay (days)^a^	51.00 (34.00, 87.00)	50.00 (37.00, 72.00)	0.341	0.733
Length of stay in ICU^a^	16.00 (0.00, 36.75)	8.00 (0.00, 23.50)	2.222	0.026
Solid tumor	54 (39.13%)	23 (36.51%)	0.126	0.723
Diabetes	31 (22.46%)	9 (14.29%)	4.721	0.030
Pancreatitis^b^	16 (11.59%)	5 (0.79%)	0.618	0.432
Total parenteral nutrition	108 (78.26%)	53 (84.13%)	0.934	0.334
Renal failure	23 (16.67%)	7 (11.11%)	1.051	0.035
Recent surgery (within 2 weeks)	60 (43.48%)	30 (47.62%)	0.300	0.584
Use immunosuppressants within the past 30 days^b^	9 (6.52%)	4 (6.35%)	–	1.000^b^
Stay in ICU during hospitalization	101 (73.19%)	39 (61.90%)	2.605	0.107
Hypoproteinemia	95 (68.84%)	38 (60.32%)	1.404	0.236
Invasive mechanical ventilation	94 (68.11%)	37 (58.73%)	1.679	0.195
Urinary catheter	121 (87.68%)	50 (79.37%)	2.356	0.125
Gastric tube	96 (69.57%)	43 (68.25%)	0.035	0.852
Central venous catheter	106 (76.81%)	49 (77.78%)	0.022	0.880
Drainage catheter	99 (71.74%)	44 (69.84%)	0.076	0.783
Septic shock	34 (24.64%)	15 (23.81%)	0.016	0.899
Multiple hospitalizations within 2 years (> 2 times)	106 (76.81%)	49 (77.78%)	1.647	0.199
Serum albumin level^a^ (g/L)	27.55 (23.45, 30.88)	28.50 (24.33, 32.80)	− 1.030	0.302
Serum creatinine level^a^ (μmol/L)	59.00 (38.00, 91.00)	54.50 (43.25, 84.75)	0.188	0.851
Leukocyte count^a^ (10^9^/L)	8.73 (6.37, 11.65)	7.13 (4.34, 10.25)	2.519	0.012
Total bilirubin level^a^ (μmol/L)	14.35 (9.28, 28.48)	15.30 (10.55, 27.95)	− 0.891	0.373
Neutrophil count^a^ (10^9^/L)	7.02 (4.65, 9.89)	5.07 (3.32, 8.25)	2.771	0.006
Lymphocyte count^a^ (10^9^/L)	0.89 (0.62, 1.21)	0.75 (0.54, 1.24)	1.399	0.162
CRP^a^ (mg/mL)	97.40 (66.20, 137.00)	110.50 (64.85, 135.5)	− 0.498	0.619
PCT^a^ (ng/mL)	0.57 (0.26, 1.75)	0.51 (0.29, 2.74)	− 0.387	0.699

^a^Is described by median and quartile, and the statistic was the Z value; other items were described as numbers (n—%) and the statistic was the χ^2^ value

^b^Statistic was the Fisher χ^2^ value

### Analysis of risk factors in patients with single and multiple candidal infections

Of the 246 patients with both *Candida* and bloodstream infections, 70 (28.45%) had multi-candidal infection and 176 (71.55%) patients had single candidal infection, and the demographic and clinical characteristics of patients are shown in Table 3.

**Table 3:** Analysis of risk factors in patients with single candidal infection and multiple candidal infections

	Single candidal infection (N = 176)	Multiple candidal infection (n = 70)	Statistic	*P value*
Male	121 (68.75%)	38 (54.29%)	4.584	0.032
Age (years)^a^	63.00 (54.00, 74.00)	65.00 (53.00, 76.50)	− 1.024	0.306
Length of stay (days)^a^	42.00 (28.00, 66.25)	57.00 (37.50, 97.75)	− 3.338	< 0.001
Length of stay in ICU^a^	7.00 (0.00, 20.00)	22.50 (6.00, 61.75)	− 4.410	< 0.001
Solid tumor	74 (42.05%)	22 (31.43%)	2.372	0.124
Diabetes	25 (14.20%)	22 (31.43%)	9.613	0.002
Pancreatitis^b^	20 (11.36%)	5 (7.14%)	0.977	0.323
Total parenteral nutrition	139 (78.98%)	57 (81.43%)	0.186	0.666
Renal failure	23 (13.07%)	13 (18.57%)	1.214	0.271
Recent surgery (within 2 weeks)	91 (51.70%)	21 (30.00%)	9.513	0.002
Use immunosuppressants within the past 30 days^b^	14 (7.95%)	1 (1.43%)	–	0.074^b^
Stay in ICU during hospitalization	114 (64.77%)	54 (77.14%)	3.539	0.060
Hypoproteinemia	125 (71.02%)	44 (62.86%)	1.553	0.213
Invasive mechanical ventilation	103 (58.52%)	50 (71.43%)	3.548	0.060
Urinary catheter	146 (82.95%)	64 (91.43%)	2.879	0.090
Gastric tube	116 (65.91%)	50 (71.43%)	0.695	0.404
Central venous catheter	127 (72.16%)	58 (82.86%)	3.074	0.080
Drainage catheter	116 (65.91%)	52 (74.29%)	1.623	0.203
Septic shock	35 (19.89%)	23 (32.86%)	4.676	0.031
Multiple hospitalizations within 2 years (> 2 times)	115 (65.34%)	52 (74.29%)	1.838	0.175
Persistent fungal infection	84 (47.73%)	54 (77.14%)	21.671	< 0.001
Serum albumin level^a^ (g/L)	27.00 (22.70, 30.70)	28.2 (24.18, 31.90)	− 1.579	0.114
Serum creatinine level^a^ (μmol/L)	60.00 (43.00,88.00)	58.00 (35.25, 91.50)	1.007	0.314
Leukocyte count^a^ (10^9^/L)	8.32 (5.69, 11.60)	8.45 (6.00, 10.91)	− 0.245	0.806
Total bilirubin level^a^ (μmol/L)	15.40 (9.50, 31.13)	13.50 (9.53, 26.05)	1.261	0.207
Neutrophil count^a^ (10^9^/L)	6.68 (4.42, 9.96)	6.43 (4.36, 8.43)	0.599	0.549
Lymphocyte count^a^ (10^9^/L)	0.75 (0.53, 1.10)	1.02 (0.75, 1.32)	− 3.127	0.002
CRP^a^ (mg/mL)	110.00 (70.83, 169.25)	93.1 (34.93, 124.50)	2.196	0.028
PCT^a^ (ng/mL)	0.86 (0.27, 4.49)	0.44 (0.25, 1.19)	2.257	0.024

^a^Is described by median and quartile, and the statistic was the Z value; other items were described as numbers (n—%) and the statistic was the χ^2^ value,

^b^Statistic was the Fisher χ^2^ value

Furthermore, the duration of hospital and ICU stays was longer in patients with multi-candidal infection than in patients with single candidal infection (hospital stay: 57 versus 42 days, respectively, based on the median; ICU stay: 22.5 versus 7 days, respectively, based on the median). In addition, patients with multi-candidal infection were more likely to have diabetes and develop septic shock. Furthermore, more than half (51.70%, 91/176) and approximately one-third (30%, 21/70) of post-surgical patients had multi-candidal infection. Moreover, when infected patients (not only post-surgical patients) were considered, 77.14% (84/176) patients with multi-candidal infection and 47.73% (54/70) patients with single candidal infection developed persistent infection, with increased CRP and PCT levels. The lymphocyte count was distinctly reduced in patients with single candidal infection (0.75 × 10^9^/L based on the median) but only slightly reduced in patients with multi-candidal infection (1.02 × 10^9^/L based on the median). Differences were statistically significant.

### Prediction of risk factors of death using machine learning

We used random forest, logistic regression and support-vector machine algorithms to develop a prediction model, and the performance evaluation is shown in Table 4.

**Table 4:** Performance of the machine-learning algorithms

Model	Accuracy	Precision	Recall	F1	AUC
Logistic regression (LR)	0.716	0.559	0.760	0.644	0.753
Random forest (RF)	0.784	0.622	0.920	0.742	0.919
Support vector machine (SVM)	0.622	0.465	0.800	0.588	0.777

**Fig. 2:**
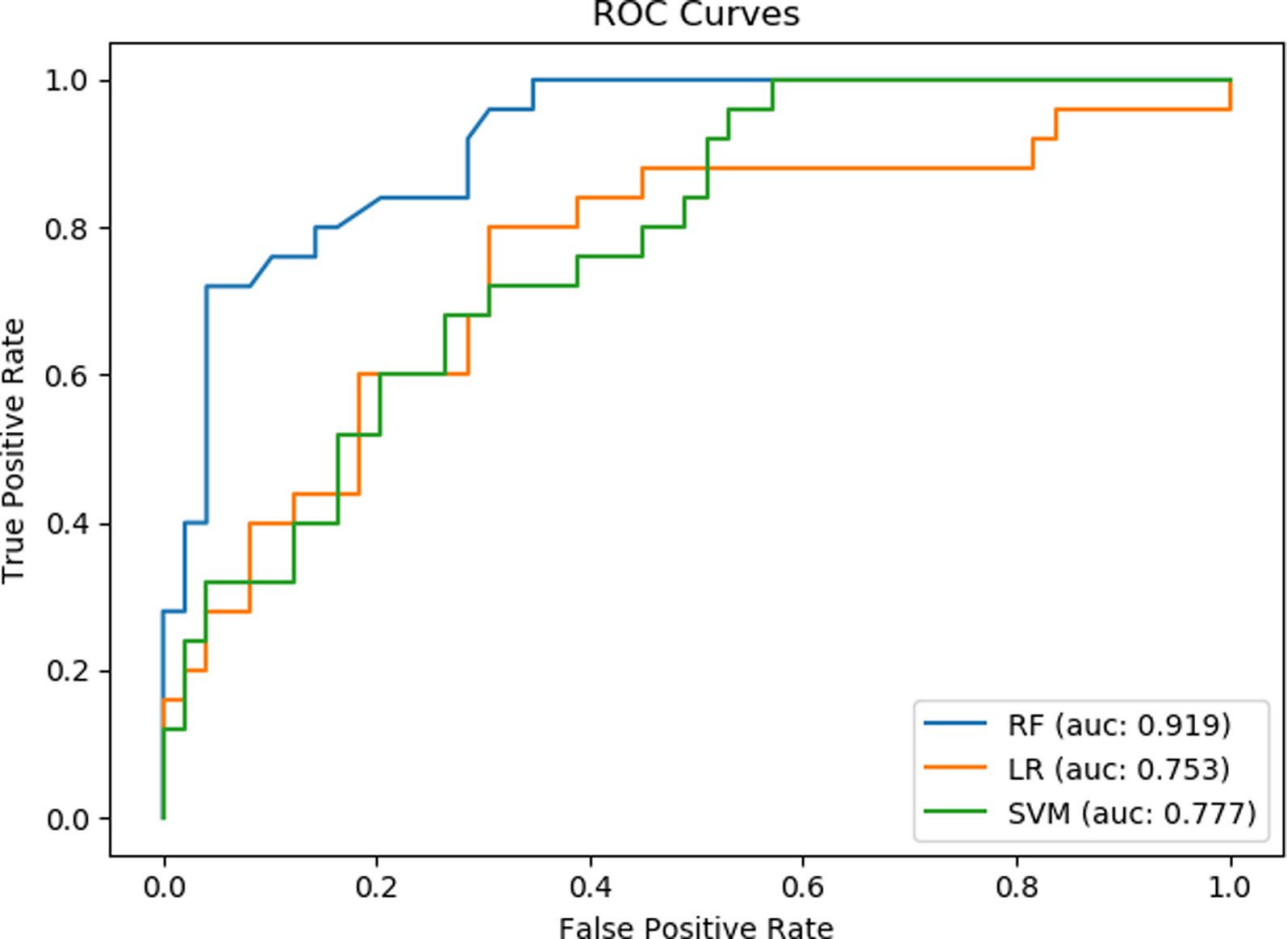
Receiver operating characteristic curve of different machine learning models. *LR* logistic regression, *RF* random forest, *SVM* support vector machine

Figure 2 demonstrates the ROC curves of the prediction model. Based on analysis and training, it was found that the random forest model exhibited the best performance. A random forest model is usually used to examine the importance of different features. The most predictive characteristics of invasive candidal infection concomitant with bacterial bloodstream infection were identified to be serum creatinine, serum albumin, CRP, PCT and total bilirubin levels; age; length of stay in the hospital; stay in ICU during hospitalisation and leukocyte and neutrophil counts (Table 5).

**Table 5:** Feature importance rank

Risk factor variables	Importance rank
Serum creatinine level	0.110647
Age	0.093094
Length of stay (days)	0.079532
Stay in ICU during hospitalization	0.066953
Serum albumin level	0.059160
CRP	0.057764
Leukocyte count	0.054652
Neutrophil count	0.054205
PCT	0.052429
Total bilirubin level	0.052185

## Discussion

To date, there have been a few epidemiological studies on patients with concomitant invasive candidal infection and bacterial bloodstream infections. We included 246 patients with concomitant invasive candidal infection and bacterial bloodstream infections admitted to a provincial medical centre in northeast China between January 2013 and January 2018. Using machine learning techniques, we found that the main predictors of death were serum creatinine, serum albumin, CRP, PCT and total bilirubin levels; age; length of stay in the hospital; stay in ICU during hospitalisation and leukocyte and neutrophil counts. The random forest model with these 10 features showed satisfactory performance, and the AUC value in the training and test sets was 0.919.

Furthermore, the epidemiological survey revealed that 96.7% (238/246) patients were hospitalised for more than 10 days, and 68.3% (168/246) patients were admitted to ICU. Most patients had multiple admissions in the past 2 years (167/246, 67.9%) and had hypoproteinaemia (169/246, 68.7%). These conditions reflect the physical characteristics of patients, which are similar to those reported in recent studies [17, 18]. Other common causes of *Candida* infections included the use of urinary catheter (210/246, 85.4% patients), central venous catheter (185/246, 75.2% patients), gastric tube (166/246, 67.5% patients), drainage catheter (168/246, 68.3% patients), invasive mechanical ventilation (153/246, 62.2% patients) and total parenteral nutrition (196/246, 79.7% patients), suggesting that infections may be associated with invasive medical operations, especially owing to long-term catheter retention. Similar results have been reported in recent studies as well [19–26].

*Candida parapsilosis* was the most common causative fungal agent (92/246, 37.4% patients), followed by *Candida guilliermondi* (53/246, 21.5% patients), *Candida albicans* (49/246, 19.9% patients), *Candida glabrata* (26/246, 10.6% patients), *Candida tropicalis* (18/246, 7.3% patients) and *Candida krusei* (4/246, 1.6% patients). Furthermore, *Acinetobacter baumannii* (111/246, 45.1% patients) was the most common causative agent of bacterial bloodstream infection, followed by *Enterococcus faecium* (72/246, 29.3% patients), *Pseudomonas aeruginosa* (65/246, 26.4% patients), *Escherichia coli* (55/246, 22.4% patients) and *Klebsiella pneumoniae* (46/246, 18.7% patients). A total of 28 types of bacteria were cultured, with Gram-positive bacteria being the main pathogenic bacteria (15/28, 53.6%). In addition, the results indicated that the rate of *Candida* infection differed according to regions, which is an important factor that should be studied further [27, 28].

In this study, the main predictors of death were serum creatinine, serum albumin, CRP, PCT and total bilirubin levels; age; length of stay in the hospital; stay in ICU during hospitalisation and leukocyte and neutrophil counts. High serum creatinine level is a risk factor for bacterial bloodstream infection and may be associated with renal insufficiency [29, 30]. In addition, age is a significant prognostic risk factor for nosocomial infections, and elderly patients are more likely to present with underlying diseases, low immunity and decreased organ function, which makes them more susceptible to invasive candidal infection/bacterial bloodstream infection [31, 32]. The length of stay in the hospital is an important index influenced by many factors, including the demographic characteristics, treatment complexity, complications and discharge plan of patients, and can be used as a predictor of death [33, 34]. Studies have shown that the overall mortality rate of hospitalised patients increases with the increasing duration of ICU stay, possibly owing to complications resulting from long-term intensive care [35, 36]. In addition, serum albumin level is a nutritional index and an important indicator of morbidity and mortality in critically ill patients. Low serum albumin level is an important and unique predictor of mortality [37, 38]. CRP is a classic indicator of infection. Previous studies have shown that CRP can also be used as a prognostic indicator for hospitalised patients [39, 40]. In addition, this study shows that increased leukocyte counts indicate increased mortality in hospitalised patients with infection. Similar studies have shown that the death rate of patients with cancer and dengue increases with increasing leukocyte counts [41, 42]. Infections destroy the dynamic balance of the immune system and cause significant changes in the neutrophil count, which are closely related to mortality [43, 44]. Furthermore, PCT is a classic indicator of infection, and recent studies have shown that PCT can also be used as a prognostic indicator for hospitalised patients with infection [45, 46]. In addition, total bilirubin levels can be used as a prognostic indicator in patients with coronavirus infection, respiratory tract infection and cardiogenic shock, and increased serum bilirubin levels are independently associated with mortality [47–49].

However, this study had some limitations. First, this is a single-centre study. Therefore, the results and conclusions may be affected by geographical location, hospital management strategies, infection control policies and susceptibility models. Second, owing to a retrospective design, some key factors of concomitant invasive candidal infection and bacterial bloodstream infections may have been ignored. In addition, to the best of our knowledge, machine learning was used for the first time in this study to predict the risk factors of death and prognosis of concomitant invasive candidal infection and bacterial bloodstream infections. Moreover, the relatively small sample size may affect the credibility of the results. Therefore, further large-scale, multi-centre prospective studies should be conducted to validate the results of this study.

## Conclusion

The most common *Candida* and bacterial species in patients with concomitant *Candida* and bacterial bloodstream infections in the First Hospital of the China Medical University were *Candida parapsilosis* and *Acinetobacter baumannii*, respectively. The main predictors of death were serum creatinine, serum albumin, CRP, PCT and total bilirubin levels; age; length of stay in the hospital; stay in ICU during hospitalisation and leukocyte and neutrophil counts.

## Supplementary Information

**Additional file 1.** Detailed data of patients with invasive Candida infection complicated with bacterial bloodstream infection.

**Additional file 2.** Detailed data on bacterial species in patients with bacterial bloodstream infection.

**Additional file 3.** Detailed data of drug sensitivity results.

## Data Availability

The data supporting the findings of this study from the corresponding author upon request. If someone wants to request the data from this study, please contact Xiuhao Guan.
